# Yet More Evidence of Collusion: a New Viral Defense System Encoded by *Gordonia* Phage CarolAnn

**DOI:** 10.1128/mBio.02417-18

**Published:** 2019-03-19

**Authors:** Matthew T. Montgomery, Carlos A. Guerrero Bustamante, Rebekah M. Dedrick, Deborah Jacobs-Sera, Graham F. Hatfull

**Affiliations:** a Department of Biological Sciences, University of Pittsburgh, Pittsburgh, Pennsylvania, USA; Carnegie Mellon University

**Keywords:** bacteriophage, *Gordonia*, viral defense

## Abstract

Temperate phages play important roles in the physiology of their bacterial hosts and establish a lysogenic relationship with the host through which prophage-expressed genes confer new phenotypes. A key phenotype is prophage-mediated defense against heterotypic viral attack, in which temperate phages collude with their bacterial host to prevent other phages from attacking, sometimes with exquisite specificity. Such defense systems have been described in *Pseudomonas* and *Mycobacterium* phages but are likely widespread throughout the microbial community. Here, we describe a novel prophage-mediated defense system encoded by *Gordonia* phage CarolAnn, which defends against infection by unrelated phages grouped in cluster CZ. CarolAnn genes *43* and *44* are coexpressed with the repressor and are necessary and sufficient to confer defense against phage Kita and its close relatives. Kita and these relatives are targeted through Kita gene *53*, a gene that is of unknown function but which is the location of defense escape mutations that overcome CarolAnn defense. Expression of Kita gene *53* is toxic to Gordonia terrae in the presence of CarolAnn genes *43* and *44*, suggesting that defense may be mediated by an abortive infection type of mechanism. CarolAnn genes *43* and *44* are distant relatives of mycobacteriophage Sbash genes *31* and *30*, respectively, which also confer viral defense but use a different targeting system.

## Abstract (precis)

Prophage-mediated viral defense systems play a key role in microbial dynamics, as lysogeny is established relatively efficiently, and prophage-expressed genes can strongly inhibit lytic infection of other, unrelated phages. Demonstrating such defense systems in Gordonia terrae suggests that these systems are widespread and that there are a multitude of different systems with different specificities for the attacking phages.

## INTRODUCTION

The dynamic interactions between bacteriophages and their bacterial hosts are central to microbial evolution, with strong selection for bacterial resistance to viral infection and with phage coevolution to counter resident defense or to exploit new hosts (1–3). Although a multitude of bacterially encoded phage defense mechanisms have been described previously (4–9), recent reports showed that some *Pseudomonas* phages and some *Mycobacterium* phages also encode viral defense systems (10, 11). These are expressed from prophages in lysogenic strains and are often heterotypic—i.e., they often defend against unrelated phages—and can be highly specific for a particular subset of attacking phages (10). Because temperate phages are common and because a large proportion of sequenced bacterial genomes carry at least one prophage, such defense systems may be widespread.

Resistance to phage infection can be mediated by mutation of the phage receptor, interruption of DNA injection (12), or abortive infection following DNA injection, preventing production of infectious progeny (8). Such abortive infection (abi) systems are widespread (8) and can be mediated by toxin-antitoxin (TA) systems that lead to cell death under conditions of infection by particular phages (9, 13). Phage lambda codes for another two-gene system in which *rexA* and *rexB* genes confer exclusion against T4*rII* mutants, such that T4*rII* infection results in depolarization of the membrane and interruption of macromolecular biosynthesis and altruistic cell death (14–16). The phage defense systems encoded by the cluster N mycobacteriophages are typically active against heterotypic phages and likely encompass a variety of mechanisms (10). For example, the Charlie prophage expresses a single membrane protein that excludes DNA injection by phage Che9c, whereas the systems encoded by prophages of Phrann and MichelleMyBell are typical of abortive infection systems, where phage adsorption and DNA injection proceeds but phage production is thwarted (10).

*Gordonia* spp. are ubiquitous mildly acid-fast nocardioform bacteria classified within the order *Actinomycetales* (17). *Gordonia* species are important components of wastewater treatment systems (18) and are notable due to their extensive abilities with respect to biodegradation (19–21) and their propensity to be opportunistic pathogens (22–24).

*Gordonia* spp. are closely related to the mycobacteria but are generally nonpathogenic and are widespread throughout the environment. They are of interest because of their potential in industrial and environmental microbiology, with capacities for synthesis and biodegradation of particular chemical compounds, including rubber (17, 25–27). However, there are few genetic tools available, and those that are available are mostly restricted to pRC4-derived plasmids and some expression systems (28–30). A large number of phages infecting *Gordonia* hosts have been reported, and these span a considerable range of genetic diversity (31–36). More than 1,000 individual phages have been isolated, and over 300 have been sequenced (https://phagesdb.org) (37), 167 of which are currently fully annotated. These are grouped into 32 clusters according to overall genetic relatedness, and another 13 are singletons with no close relatives (31, 38). Nine of the clusters are divided into subclusters (e.g., CR1, CR2, CR3, etc.) reflecting subgroups with shared genomic characteristics. All of these clusters contain only phages that infect *Gordonia* hosts, with the notable exception of cluster A, a large group of actinobacteriophages—primarily mycobacteriophages—divided into 20 subclusters; one of these subclusters (A15) contains *Gordonia* phages (31). In addition to providing insights into phage diversity and evolution, *Gordonia* phages may be useful for the development of genetic tools for *Gordonia*, including vector systems such as integrating and low-copy-number plasmids, selectable markers, and expression systems. A similar approach has been useful for advancing mycobacterial genetics (39).

A majority of *Gordonia* phages are temperate, and many of the sequenced phages have genomic features common to temperate phages, including those in clusters/subclusters A15, CQ, CU, CW, CY, CZ, CV, CX, DB, DC, DH, DI, DL, DM, DN, and DT and singletons Catfish, Eyre, Gal1, GMA1, GMA4, Ruthy, and Yvonnetastic (31). This raises the issue of whether these might encode phage defense systems similar to those described for the cluster N mycobacteriophages (10). We note that the genome sizes of the temperate phages vary enormously, from ∼16 kbp (cluster DM) to 98.1 kbp (Yvonnetastic); the small cluster DM phages such as Emperor and SallySpecial have only 21 to 24 genes, coding for virion proteins and immunity functions, with little space to include accessory genes. However, all of the other temperate *Gordonia* phages have genomes longer than 40 kbp and genomic capacity does not constrain the ability to carry lysogenically expressed genes that contribute to viral defense.

Here we show that temperate *Gordonia* phages in cluster CV harbor prophage-expressed novel viral defense systems. Phage CarolAnn confers defense against one-third of the phages tested, with reductions of 10^−4^ or greater in plating efficiency relative to a nonlysogen. CarolAnn codes for at least two distinct defense systems, one of which is dependent on CarolAnn genes *43* and *44*, which are coexpressed with the immunity repressor in lysogeny. CarolAnn genes *43* and *44* defend against infection by cluster CZ phage Kita and other closely related phages grouped in cluster CZ. Defense by CarolAnn gp43/gp44 against phages in cluster CZ requires Kita gene *53* and its homologues, early lytic genes of unknown function. Kita DNA injection and gene expression occur normally in a CarolAnn lysogen, but phage production may be negated by the cytotoxicity of Kita gp53 under conditions of expression in the presence of CarolAnn gp43/gp44. Homologues of CarolAnn gp43/gp44 are present in mycobacteriophage Sbash but, interestingly, use a different system for target recognition and defense.

## RESULTS

### Immunity and defense by cluster CV *Gordonia* phages.

**FIG 1:**
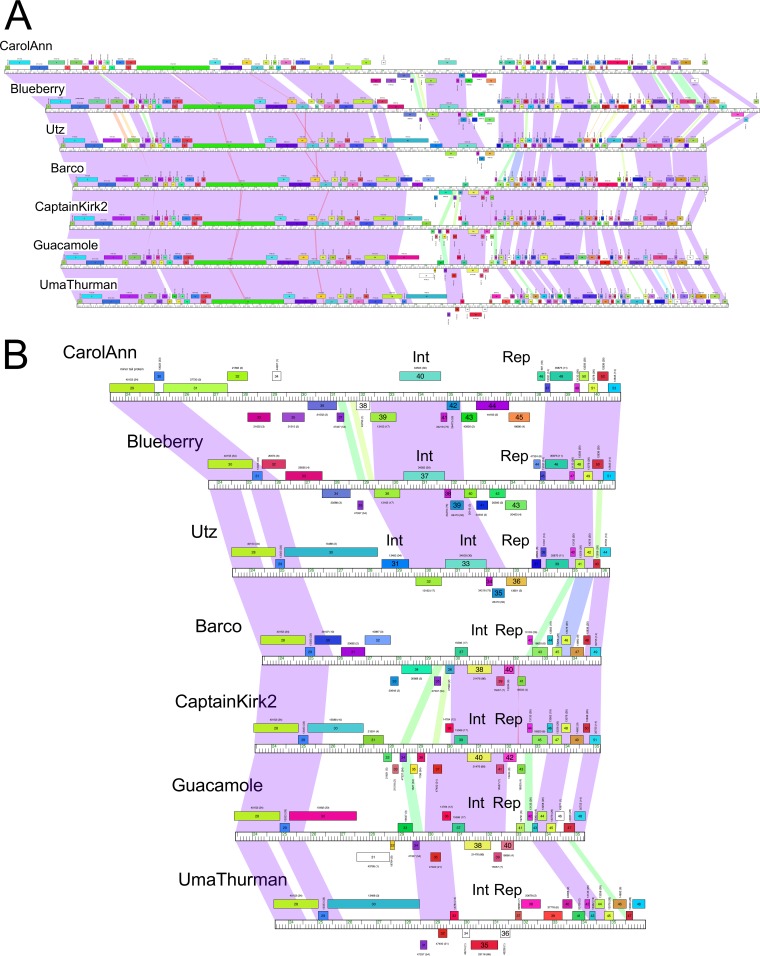
Comparisons of cluster CV *Gordonia* phages. (A) The genome maps of cluster CV phages CarolAnn, Blueberry, Utz, Barco, CaptainKirk2, Guacamole, and UmaThurman (top to bottom) are shown with spectrum-colored shading between each genome reflecting nucleotide sequence similarity (with violet representing greatest similarity and red the least similarity above a threshold E value of 10^−4^; white areas are below the threshold value). Phages shown correspond to the lysogens shown in Table 1. (B) An expanded view of the central parts of these genomes displayed as described in the panel A legend. The locations of the integrase (Int) and immunity repressor (Rep) genes are indicated. Genes are shown as colored boxes above and below genome rulers. Gene numbers are shown within the boxes, and the assigned Pham numbers (45) are shown above the boxes, with the number of Pham members shown in parentheses. Gene boxes are colored according to their Pham assignment.

Cluster CV contains seven closely related phages (Barco, Captainkirk2, Blueberry, CarolAnn, Guacamole, UmaThurman, and Utz) isolated on G. terrae 3612 (31, 32, 40). These phages are temperate and form stable lysogens that are immune to superinfection by themselves (Table 1). Alignment of the genomes showed that they share high levels of nucleotide sequence similarity in leftmost parts of their genomes containing the virion structure and assembly genes, have interrupted segments of similarity in the rightmost parts of their genomes (Fig. 1A), and have substantial variation at the centers of the genomes where the immunity and integration functions are located (Fig. 1B). A putative repressor gene coding for a helix-turn-helix DNA binding protein responsible for superinfection immunity can be readily predicted in each genome, although these putative repressors span considerable sequence diversity; only the Barco and CaptainKirk2 putative repressors are closely related (99% amino acid [aa] identity; Fig. 1B). Here we use the term “immunity” to refer to the inhibition of infection by phages with closely related repressor systems.

**TABLE 1:** Viral defense conferred by lysogens of cluster CV phages^*a*^

Phage	Cluster	GenBank accession no.	% plating efficiency for Gordonia terrae lysogenic strain 3612						
			Barco	CaptainKirk2	Blueberry	CarolAnn	Guacamole	UmaThurman	Utz
Barco	CV	MK501730	<10^−8^	<10^−8^	10^−5^	1	<10^−9^	1	1
CaptainKirk2	CV	KX557274	<10^−9^	<10^−9^	10^−5^	1	1	1	10^−6^
Obliviate	CV	KU963254	<10^−8^	<10^−8^	10^−6^	1	1	1	10^−6^
Blueberry	CV	KU998236	<10^−8^	1	<10^−8^	10^−4^	1	1	1
CarolAnn	CV	KX557275	<10^−9^	10^−3^	<10^−7^	<10^−9^	<10^−9^	1	10^−7^
Guacamole	CV	KU963259	<10^−8^	10^−4^	10^−5^	1	<10^−9^	1	1
UmaThurman	CV	KU963251	10^−4^	10^−3^	10^−4^	10^−3^	1	<10^−9^	1
Utz	CV	KU998248	10^−5^	1	1	10^−4^	1	1	<10^−9^
Walrus	CV	MK501729	1	1	10^−6^	1	1	1	1
Rosalind	A15	KU998250	1	1	1	1	1	1	1
Soups	A15	KU998249	1	1	1	1	1	1	1
Strosahl	A15	KX557284	1	1	1	1	1	1	1
KatherineG	A15	KU998251	1	1	1	1	1	1	1
Bachita	CQ1	KU998247	1	1	<10^−7^	<10^−8^	1	1	1
OneUp	CQ2	KU998245	1	1	1	<10^−8^	1	1	1
ClubL	CQ1	KU998246	1	1	<10^−7^	<10^−8^	1	1	1
Kabluna	CR	MF919510	1	1	1	1	1	1	1
Monty	CS2	KU998241	1	1	1	10^−5^	1	1	1
Woes	CS3	KU998240	1	1	1	10^−5^	1	1	1
Kvothe	CS4	KU998243	1	1	1	1	1	1	1
Emalyn	CT	KU963260	1	1	1	1	1	1	1
Cozz	CT	KU998239	1	1	1	1	1	1	1
Huffy	CU1	KY471268	1	1	1	1	1	1	1
Vendetta	CU1	KU998237	1	1	1	1	1	1	1
Orchid	CX	KU998253	1	1	1	1	1	1	1
Kampe	CX	KU998254	1	1	1	1	1	1	1
PatrickStar	CX	KU998252	1	1	1	1	1	1	1
BritBrat	CY2	KU998233	10^−5^	1	1	1	<10^−7^	1	1
Bialota	CZ1	MK016492	1	1	<10^−8^	10^−5^	1	1	10^−6^
Kita	CZ1	KU963257	1	1	1	10^−5^	1	1	10^−6^
Batstarr	CZ1	KX557273	1	1	10^−5^	10^−5^	1	1	10^−3^
Nymphadora	CZ1	KU963255	1	1	<10^−8^	10^−5^	1	1	1
SoilAssassin	CZ2	KU963246	1	1	10^−3^	10^−6^	1	1	10^−5^
Ebert	CZ2	MH271295	1	1	10^−5^	10^−7^	1	1	1
Yeezy	CZ3	KU963249	1	1	1	10^−3^	1	1	1
Howe	CZ4	KU252585	1	1	10^−4^	1	1	1	1
Bowser	DB	KU998235	<10^−7^	10^−3^	1	1	<10^−8^	1	1
Wizard	DC	KU998234	1	1	1	<10^−8^	1	1	10^−3^
Phinally	DE2	KU963253	1	1	1	1	1	1	1
Terapin	DG	KX557285	1	1	1	1	1	1	1
Betterkatz	DI	KU963261	1	1	10^−6^	1	1	1	1
Emperor	DM	MH271296	1	1	1	1	1	1	1
Yvonnetastic	Sin	KU963248	1	1	1	1	1	1	1

a Data represent efficiencies of plating relative to infection of a nonlysogenic strain of Gordonia terrae 3612.

**FIG 2:**
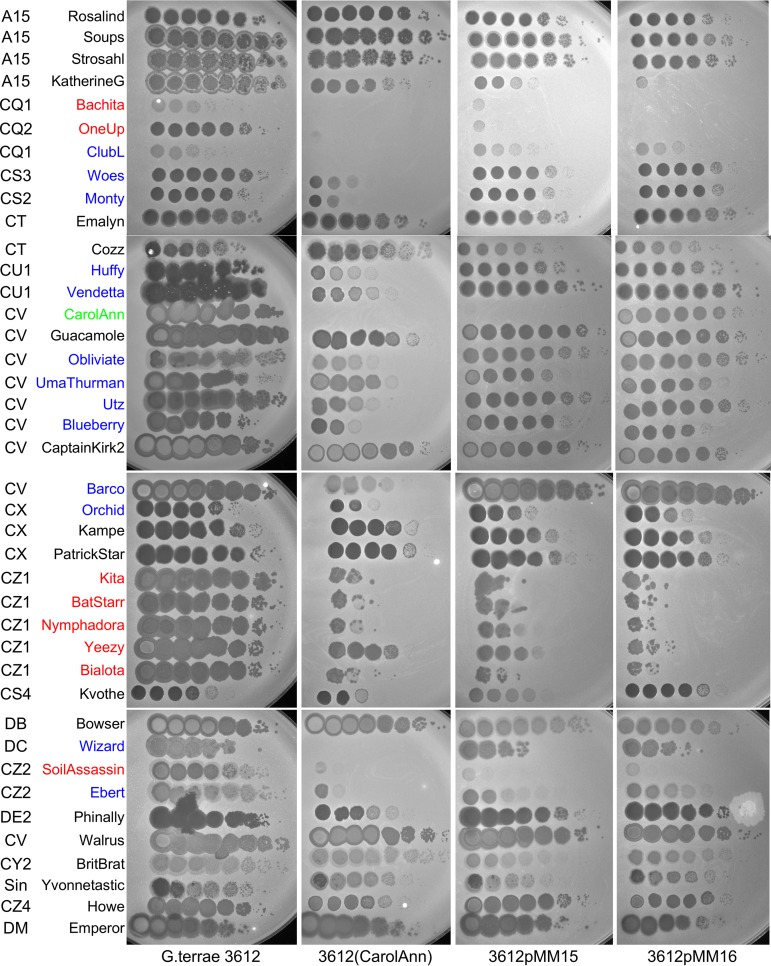
Plating efficiencies of *Gordonia* phages and defense patterns. Ten-fold serial dilutions of the phages indicated on the left were plated onto lawns of G. terrae 3612, a CarolAnn lysogen [3612(CarolAnn)], 3612pMM15, and 3612pMM16 (see Fig. 3). Phage names shown in red correspond to those that have markedly reduced plating efficiencies on the CarolAnn lysogen as well as the pMM15 and pMM16 strains. Phage names in blue correspond to those that plate with reduced efficiencies on the CarolAnn lysogen but not on the pMM15 and pMM16 strains. CarolAnn, shown in green, is subject to repressor-mediated immunity on both the lysogen and pMM15 but not on the pMM16 strain (see Table 1).

Lysogens of each of the seven cluster CV phages were constructed in G. terrae 3612 and tested for immunity against each of the others, as well as against two additional cluster CV phages, Obliviate and Walrus (Table 1) (Fig. 2). However, the immunity patterns are somewhat complex, and although the lysogens show strong superinfection immunity to themselves, they show no immunity to other CV phages (e.g., UmaThurman), reciprocal homoimmunity (e.g., CaptainKirk2 and Barco), nonreciprocal immunity (e.g., CarolAnn and Guacamole), or reductions in immunity in plating by 3 to 5 orders of magnitude. It is likely that the strong (e.g., 10^−7^ or greater) reductions in infectivity are repressor mediated and that the more modest reductions result from the presence of other defense systems from which mutational escape has occurred, as observed in some other defense systems (10).

We also tested the seven lysogens for susceptibility to infection by 34 additional *Gordonia* phages broadly spanning the diversity of the sequenced phages (Table 1). With the exception of UmaThurman, all of the lysogens confer strongly reduced plating efficiencies to at least one heterotypic phage (Table 1). In some instances (e.g., infection of CarolAnn by Wizard) (Table 1) (Fig. 2), the reduction in plating was large and no plaque formation was observed even at the highest phage titer. In other cases, plating was reduced substantially (by 5 to 6 orders of magnitude) but incompletely (e.g., infection of CarolAnn by Kita) (Table 1) (Fig. 2). CarolAnn conferred reduced plating to about one-third of the tested phages, especially those in clusters CQ, CS, CU, CZ, and DC, although for some phages (e.g., Huffy, KatherineG, Guacamole), the differences were reflected more by a strong diminution in plaque size rather than by plaque formation *per se* (Fig. 2). Because all of the cluster CV lysogens differ in their defense patterns, we predict that the variable set of genes located near the centers of the genomes (Fig. 1B) are likely to be involved in conferring these phenotypes.

### Lysogenic expression of CarolAnn, Blueberry, and Utz prophages.

**FIG 3:**
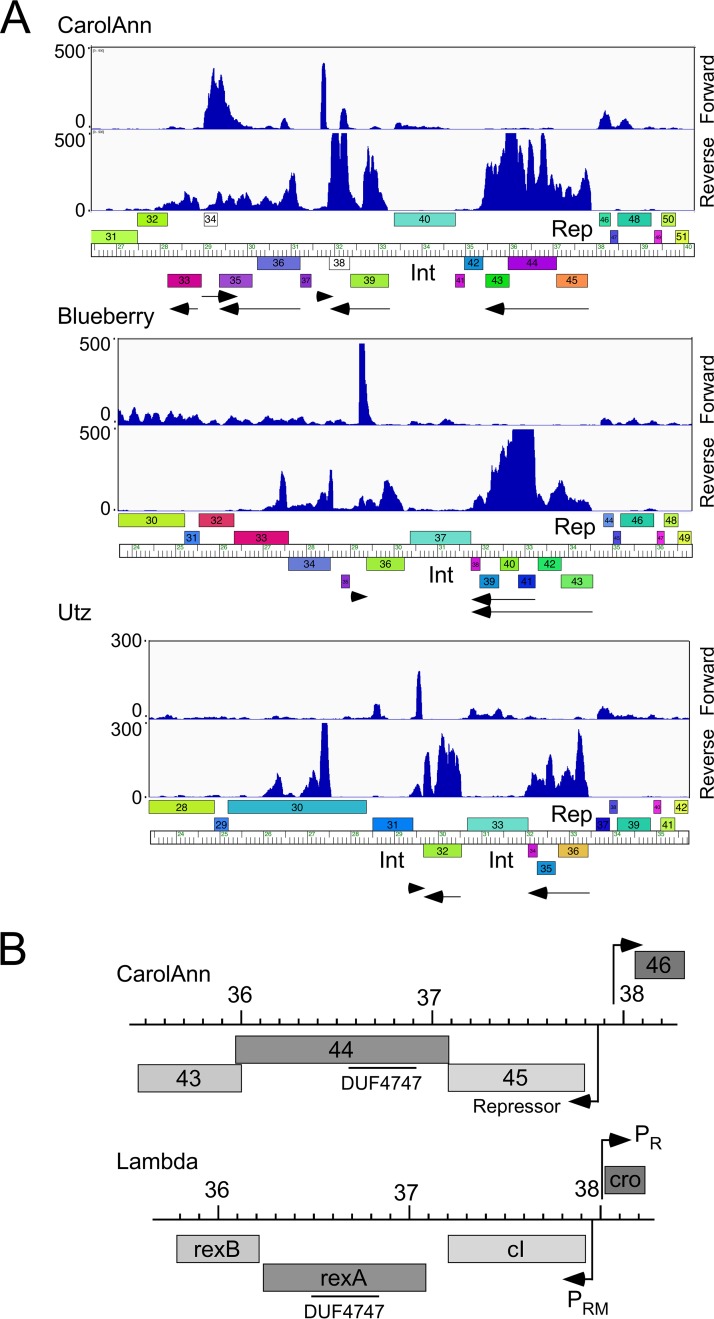
Transcriptomic patterns of cluster CV lysogens. (A) RNA was isolated from G. terrae 3612 lysogens of CarolAnn, Blueberry, and Utz and analyzed by RNAseq. The central portions of the genome are shown; the full genome profiles are in Fig. S1 to S3. The forward and reverse strand reads are shown scaled to the map of the phage genome indicated at the bottom. Arrows below the map indicate the transcribed regions. Gene boxes are colored according to their Pham assignment; colors reflect sequence similarity. Integrase (Int) and repressor genes (Rep) are indicated. (B) Comparison of CarolAnn and lambda genomes. Segments of the two phage genomes are aligned to show similarities between CarolAnn genes *43* to *46* and lambda exclusion genes *rexA*, *rexB*, *cI*, and *cro*. The positions of lambda promoters P_R_ and P_RM_ are shown as well as those of putative CarolAnn promoters. The locations of a DUF4747 conserved domain in CarolAnn gp44 and lambda RexA are indicated. CarolAnn gp46 is likely a *cro*-like protein. Genome coordinates (indicated in kilobase pairs) are shown centrally.

To determine which prophage genes are expressed, RNA was isolated from lysogenic cultures of CarolAnn, Blueberry, and Utz and was analyzed by transcriptome sequencing (RNAseq) (Fig. 3A; see also Fig. S1, S2, and S3 in the supplemental material). Lysogens of CarolAnn, Blueberry, and Utz, as expected, showed expression of the immunity repressor (Fig. 3A) as well as expression of several closely linked genes; a low level of lytic genes was also observed in some cultures (Fig. S1, S2, and S3). In the three lysogens, 1 to 5 genes closely linked to the repressor were expressed, most of which are of unknown function. In CarolAnn, these included genes *43* and *44*, which are cotranscribed leftward with the repressor gene (gene *45*), with leftward-transcribed genes *38* and *39*, and at somewhat lower levels, with genes *34* to *36* (Fig. 3A). An approximately 150-bp noncoding region was also transcribed rightward immediately to the left of gene *38*. The Blueberry and Utz lysogens similarly show expression of genes closely linked to their repressors, together with several other genes in this region (Fig. 3A). Thus, genes such as the homologues Blueberry *36*, Utz *32*, and CarolAnn *39* may also be involved in phage defense.

10.1128/mBio.02417-18.1 FIG S1 RNAseq profile of CarolAnn gene expression in a CarolAnn lysogen of G. terrae 3612. RNAseq reads obtained from a CarolAnn lysogen were mapped to the viral orientation of the CarolAnn genome. Forward-strand and reverse-strand reads are shown in the upper and lower panels, respectively. Download FIG S1, PDF file, 0.5 MB. Copyright © 2019 Montgomery et al. 2019 Montgomery et al. This content is distributed under the terms of the Creative Commons Attribution 4.0 International license.

10.1128/mBio.02417-18.2 FIG S2 RNAseq profile of Blueberry gene expression in a Blueberry lysogen of G. terrae 3612. RNAseq reads obtained from a Blueberry lysogen were mapped to the viral orientation of the Blueberry genome. Forward-strand and reverse-strand reads are shown in the upper and lower panels, respectively. Download FIG S2, PDF file, 0.6 MB. Copyright © 2019 Montgomery et al. 2019 Montgomery et al. This content is distributed under the terms of the Creative Commons Attribution 4.0 International license.

10.1128/mBio.02417-18.3 FIG S3 RNAseq profile of Utz gene expression in a Utz lysogen of G. terrae 3612. RNAseq reads obtained from a Utz lysogen were mapped to the viral orientation of the Utz genome. Forward-strand and reverse-strand reads are shown in the upper and lower panels, respectively. Download FIG S3, PDF file, 0.5 MB. Copyright © 2019 Montgomery et al. 2019 Montgomery et al. This content is distributed under the terms of the Creative Commons Attribution 4.0 International license.

The expression of CarolAnn genes *44* and *43* is of particular interest because these are homologues of genes *30* and *31*, respectively, of mycobacteriophage Sbash (see accompanying paper [41]). Sbash is a cluster I2 mycobacteriophage, and Sbash *30* and *31* confer defense against infection by the cluster L phage Crossroads. CarolAnn gp44 and Sbash gp30 comprise 370 and 372 residues, respectively, and share 50% amino acid identity; CarolAnn gp43 and Sbash gp31 are 178 and 165 residues, respectively, and share 42% amino acid identity; there are no other related genes in the collection of >2,800 sequenced actinobacteriophages (https://phagesdb.org). Both CarolAnn gp43 and Sbash gp31 are predicted membrane proteins, each with four putative transmembrane domains, and although there are few bioinformatic clues to the functions of CarolAnn gp44 and Sbash gp30, they both have a weak HHPred match to the conserved domain motif, DUF4747 (Fig. 3B). Interestingly, CarolAnn gp44 and Sbash gp30 have similarities to the RexAB exclusion system of phage lambda, in which RexB is a membrane protein, and RexA also has a DUF4747 domain (14, 15) (Fig. 3B). CarolAnn genes *43* and *44* are coexpressed with the repressor (gene *45*; Fig. 3A), as the *rexAB* genes are with cI in lambda (Fig. 3B). However, Sbash genes *30* and *31* are lysogenically expressed from their own promoter located upstream of gene *30*. These comparisons suggest that the CarolAnn and Sbash defense systems may function similarly to the lambda RexAB exclusion system.

### CarolAnn genes *43* and *44* confer defense against cluster CZ phages.

To characterize the roles of CarolAnn genes *43* and *44* in defense, we used two types of plasmid vectors. One type is based on the extrachromosomally replicating *oriM* gene (42) used extensively in mycobacterial genetics, and plasmids of that type efficiently transformed Gordonia terrae 3612 by the use of either hygromycin resistance or kanamycin resistance selectable markers (data not shown). The second type consists of integration-proficient plasmids carrying the *attP* site and integrase gene of mycobacteriophage L5 (e.g., pMH94), which transform by efficient and stable site-specific integration into an *attB* site overlapping a tRNA^gly^ gene (43).

**FIG 4:**
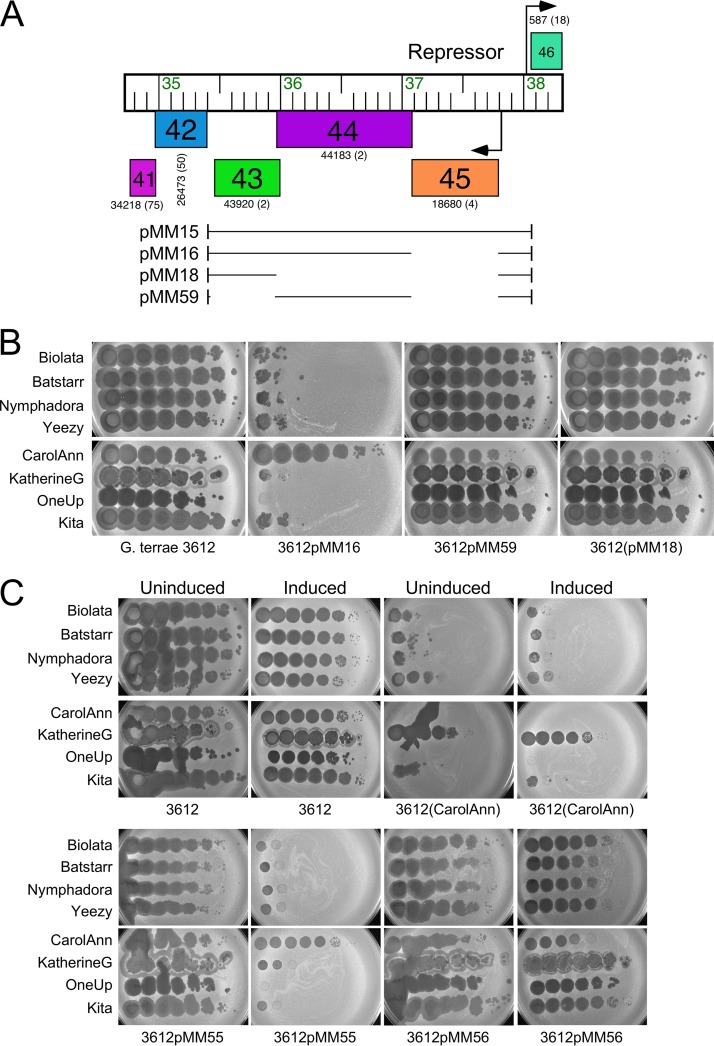
CarolAnn genes *43* and *44* confer defense against phage infection. (A) The organization of CarolAnn genes *41* to *46* is shown at the top, with arrows indicating the positions of putative promoters and the direction of transcription. Genes are represented as boxes, with the gene names within the box; the Phamily designation and the number of Pham members (in parentheses) are shown below each box. The segments of CarolAnn DNA present in plasmids pMM15, pMM16, pMM18, and pMM59 are shown as black lines. Plasmids pMM55 and pMM56 contain the same CarolAnn genes as plasmids pMM16 and pMM59, respectively, but the genes are inserted into Tet-ON vector pCCK39 (Table S2). (B) Lysates of phages Bialota, BatStarr, Nymphadora, Yeezy, CarolAnn, KatherineG, OneUp, and Kita were serially diluted 10-fold and plated onto lawns of G. terrae strains. Plasmid pMM16 confers defense against all of the phages except CarolAnn. (C) Dilutions of the same phages were plated onto lawns of G. terrae 3612 on solid medium either lacking (Uninduced) or containing (Induced) ATc inducer.

Because CarolAnn genes *43* and *44* are cotranscribed with gene *45* (Fig. 3), a DNA segment carrying all three genes and the upstream regulatory region was inserted into the integration-proficient pMH94 vector (43) to give pMM15 (Fig. 4A). Following electroporation of pMM15 into G. terrae 3612, transformants were selected and tested for phage sensitivity (Fig. 2). Plasmid pMM15 was found to confer defense against a subset of the phages that CarolAnn defends against, including CQ1 phages Bachita and OneUp and the CZ phages Kita, BatStarr, Nymphadora, Yeezy, Bialota, and SoilAssassin; it also confers immunity to CarolAnn (Fig. 2). In contrast, several phages that infect with reduced plating efficiency on the CarolAnn lysogen—Woes and Monty (CS3 and CS2, respectively); Huffy and Vendetta (CU1); UmaThurman, Utz, and Blueberry (CV); and Wizard (DC)—plate normally on the pMM15 strain.

To distinguish between the roles of the repressor gene (*45*) and genes *43* and *44*, we constructed a plasmid derivative (pMM16) in which the repressor is deleted (Fig. 4A). Plasmid pMM16 does not confer immunity to CarolAnn, confirming the identity of gene *45* as the repressor gene, but gives a profile of susceptibility similar to that determined with all other phages tested (Fig. 2 and 4B). CarolAnn genes *43* and *44* are thus implicated in reducing the plating efficiency of phages Kita, Bialota, Batstarr, Nymphadora, and Yeezy (CZ1) as well as of phages Bachita (CQ1) and OneUp (CQ2) and SoilAssassin (CZ2) (Fig. 2 and 4B), although a separate and distinct system must be involved in conferring defense against other phages such as Huffy and Vendetta (Fig. 2). Oddly, pMM15 and pMM16 confer a greater level of defense to KatherineG than the CarolAnn lysogen, a phenotype not observed with the other subcluster A15 phages (Fig. 2).

To determine if both gene *43* and gene *44* are required for defense, we constructed plasmids pMM18 and pMM59, which have gene *44* and gene *43* removed, respectively (Fig. 4A). These were transformed into G. terrae 3612 and tested for phage infection (Fig. 4B). Removal of either gene *43* or gene *44* abrogated the defense phenotype, and all of the tested phages infected efficiently. Thus, CarolAnn gp43 and gp44 are both required for defense. To demonstrate that expression *per se* of genes *43* and *44* is required, the two genes were cloned into an integration vector (pCCK39) containing a tet-inducible expression system (to make plasmid pMM55). Plasmid pMM55 conferred defense similar to that shown with the CarolAnn lysogen when induced but conferred no defense in the absence of inducer (Fig. 4C). Induction of CarolAnn gp43 and gp44 also substantially reduced the efficiency of plating of KatherineG, reflecting the phenotypes observed with pMM15 and pMM16 (Fig. 3). Removal of either gene *43* (plasmid pMM56; Fig. 4C) or gene *44* (pMM57; data not shown) resulted in loss of the defense phenotype.

### CarolAnn does not interfere with Kita lytic gene expression.

**FIG 5:**
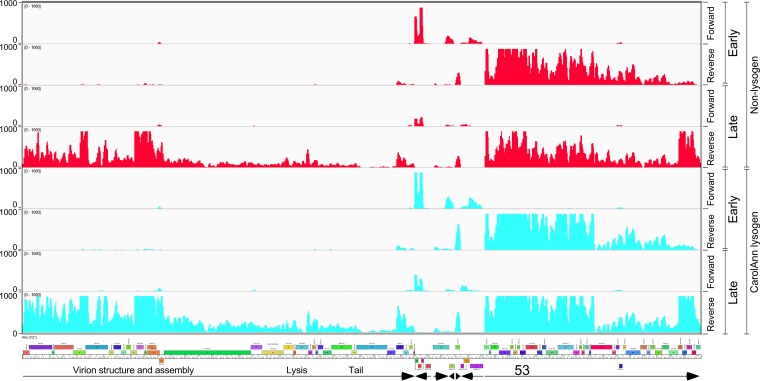
Transcriptional profile of phage Kita infection. RNA was isolated at early (30 min) and late (120 min) time points after infection of G. terrae 3612 or a CarolAnn lysogen [G. terrae 3612(CarolAnn)]—shown in red and aqua, respectively—with phage Kita; RNAseq reads were mapped to the forward and reverse strands as indicated. Arrows at the bottom indicate the regions transcribed early and late, with functional notation and with the position of Kita gene *53* shown.

Using Kita as an example of a phage targeted by CarolAnn *43*/*44* defense, we determined its lytic growth transcriptional profile using RNA isolated from infected cells at 30 min and 120 min after infection (“early” and “late” times, respectively) (Fig. 5). At the early time point, several centrally located genes (genes *36* to *38* and *41* to *45*) were expressed along with genes *46* to *79*, although RNA levels were lower at the rightward end of these genes. Notably, gene *53* was clearly transcribed during the early phase of infection, although the RNA was readily detected at the late time also. Late gene expression appeared to start near the beginning of gene *76* (Fig. 5) and to proceed through *cos* and into the virion structure and assembly genes (genes *1* to *28*). A similar profile of gene expression was observed following infection of a CarolAnn lysogen with Kita, with only minor differences from infection of a nonlysogen (Fig. 5). The CarolAnn defense system thus does not interfere with early stages of infection such as adsorption, DNA injection, or gene expression and thus presumably acts through an abortive infection system in which phage production is prevented through a block in viral assembly or cellular toxicity.

### CarolAnn gp43/gp44 defense targets Kita gp53 and its homologues.

**FIG 6:**
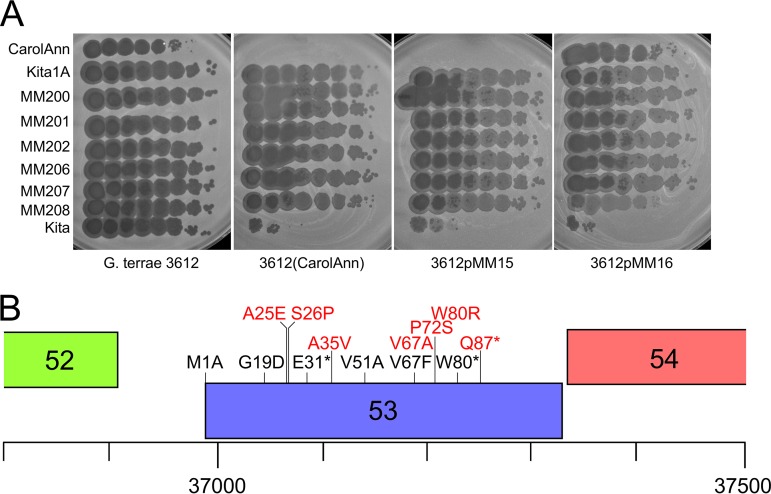
Isolation and mapping of defense escape mutants. (A) Defense escape mutants of Kita efficiently escape CarolAnn *43*/*44*-mediated defense. (B) Locations of defense escape mutant substitutions in Kita gp53 (and its homologues) are shown in black type, and the positions of nontoxic mutants are shown in red type. Of 24 independent defense escape mutants isolated, 6 introduce either termination codons or single amino acid substitutions (see Table 2). The M1A, and E31* mutants are DEMs of Kita, the G19D substitution was isolated independently in Kita and Nymphadora, the V67F substitution was identified in a Nymphadora DEM, and V51A and W80* are both DEM derivatives of Yeezy. The translation initiation codon shown for Kita *53* is at coordinate 36992 and corresponds to Kita 53-short (see the text and Fig. 7).

To determine how the CarolAnn gp43/gp44 defense system targets specific phages, we isolated defense escape mutants (DEMs) from phages Bialota, Kita, BatStarr, Nymphadora, and Yeezy (all in cluster CZ). Several individual plaques from separate lysates were recovered from infection of either a CarolAnn lysogen or a strain carrying plasmid pMM16 and were sequenced (Table 2); all of the escape mutants plated efficiently on a CarolAnn lysogen and on pMM15 and pMM16 recombinant strains (Fig. 6A). All 12 of the Kita DEMs have mutations in gene *53*, encoding a 113-residue protein of unknown function (Table 2) (Fig. 6B); two of these have an additional mutation which is present elsewhere in the genome but which presumably does not contribute to the escape phenotype. The gene *53* mutations resulted in frameshifts, small deletions and insertions, a nonsense mutant, and a change in the predicted translation start codon (Table 2) (Fig. 6B). One mutant had a single amino acid substitution, G19D. The Bialota mutants each had a single mutation in the homologue of Kita *53* (Bialota *50*); all were deletions, insertions, or frameshifts. Although the BatStarr, Nymphadora, and Yeezy DEMs had additional mutations elsewhere in the genome, they all contained changes in their homologues of Kita *53* (genes *52*, *53*, and *50*, respectively). These included an identical single amino acid substitution (G19D) with respect to the one identified in Kita *53* and two additional single amino acid substitutions, V67F and V51A (Table 2) (Fig. 6B). We note that Kita *53* homologues were present only in phages grouped in subclusters CZ1, CZ2, CZ3, and CY1. There are no bioinformatically informative clues as to their function, although they are predicted to be cytoplasmic. Although the defense system also targets Bachita and OneUp (cluster CQ1), DEMs could not be readily recovered (Fig. 2). Bachita and OneUp do not possess Kita gp53 homologues and are perhaps susceptible to CarolAnn defense through an alternative targeting mechanism.

**TABLE 2:** Defense escape mutants of cluster CZ phages

Phage	Mutant	Strain^*a*^	Mutation^*b*^	AA substitution	Notes
Bialota	Bialota 1A	pMM16	37770 ↑2 bp	gp50 f/s	
Bialota	MM100	CarolAnn	Δ37811–37833; Δ23 bp	gp50 Δ; f/s	
Bialota	MM101	CarolAnn	37671 Δ1 bp	gp50 f/s	
Bialota	MM102	CarolAnn	37625 ↑19 bp (dup)	gp50 Ins; f/s	
Bialota	MM103	CarolAnn	37712 ↑9 bp (dup)	gp50 ↑EAV	
Bialota	MM104	CarolAnn	37797 ↑24 bp (dup)	gp50 ↑ADQVAAKI	
Kita	Kita 1A	pMM16	Δ37065–37085; Δ21 bp	gp53 Δ25–31	
Kita	Kita 2E	pMM16	Δ37198–37222; Δ25 bp	gp53 Δ; f/s	
Kita	Kita 3B	pMM16	Δ37081–37089; Δ9 bp	gp53 32–34	
Kita	MM200	CarolAnn	G37082T	gp53 E31*	
Kita	MM201	CarolAnn	Δ37087–37095; Δ9 bp	gp53 Δ34–36	Also A1558T
Kita	MM203	CarolAnn	Δ37173–37196; Δ24 bp	gp53 Δ61–68	
Kita	MM204	CarolAnn	37060 ↑23 bp (dup)	gp53 Ins; f/s	
Kita	MM205	CarolAnn	Δ37087–37095; Δ9 bp	gp53 Δ34–36	Same as MM201
Kita	MM206	CarolAnn	37084 ↑9 bp (dup)	gp53 ↑VVE	
Kita	MM207	CarolAnn	G37047A	gp53 G19D	Also Δ32933–32996
Kita	MM208	CarolAnn	T36993C	gp53 M1A	
Kita	MM209	CarolAnn	52 bp Ins/Del	gp53 Ins/Del/fs	
BatStarr	BatStarr 1C	pMM16	Δ39145–39168; Δ24 bp	gp52 Δ62–69	Also A26109G
BatStarr	BatStarr 2B	pMM16	Δ39137–39160; Δ24 bp	gp52 Δ61–68	Also A26109G
Nymphadora	Nymphadora 1C	pMM16	G39010A	gp53 G19D	Also T26419C
Nymphadora	Nymphadora 2C	pMM16	G39153T	gp53 V67F	Δ24884–25969
Yeezy	Yeezy 2D	pMM16	G36133A	gp50 W80*	Also T26575C
Yeezy	Yeezy 3D	pMM16	T36045C	gp50 V51A	Also T23948C

a Defense escape mutants were isolated either on a CarolAnn lysogen or on a recombinant strain carrying pMM16.

b Arrows indicate insertions, dup refers to duplications, and Ins/Del are insertions/deletions.

This profile of mutations conferring defense escape phenotypes provides compelling evidence that Kita gp53 and its homologues are required for the CarolAnn *43*/*44* system to inhibit propagation of the superinfecting phages. It also indicates that these genes are not required for lytic growth as several of the mutations are predicted to prevent synthesis of the gene products. We note that homologues of Kita gene *53* were present only in a subset of the cluster CZ phages, as well as in some of the subcluster CY1 phages. Cluster CY1 phage Angelique has a homologue of Kita *53* and was shown to be susceptible to the CarolAnn *43*/*44* defense (data not shown), whereas BritBrat, which does not have a Kita 53 homologue, plated normally on a CarolAnn lysogen (Table 1) (Fig. 2). Likewise, subcluster CZ2 phage SoilAssassin has a Kita *53* homologue and was shown to be subject to CarolAnn *43*/*44* defense (Fig. 2), whereas CZ2 phage Ebert (a close relative of SoilAssassin), which lacks a Kita *53* homologue, was not targeted for defense by pMM15 and pMM16, although it showed reduced plating on the CarolAnn lysogen (Fig. 2).

### Kita gp53 confers CarolAnn *gp43*/*gp44*-dependent toxicity.

**FIG 7:**
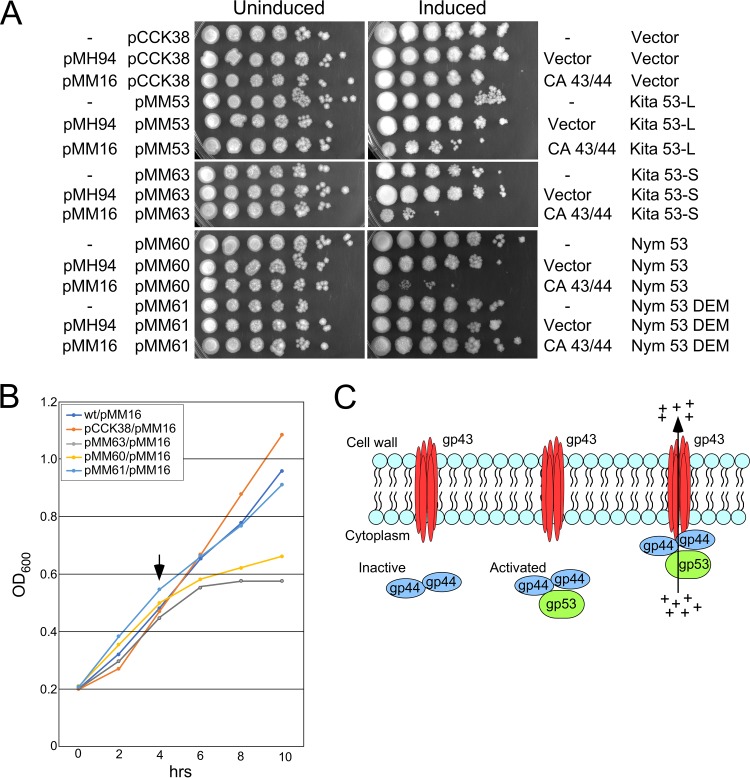
Kita gp53 is toxic in combination with CarolAnn gp43/gp44. (A) Plasmids pCCK38 (vector), pMM53 (Kita *53*-long), pMM63 (Kita *53*-short), pMM60 (Nymphadora *53*), and pMM61 (Nymphadora *53* with DEM mutation) were electroporated into G. terrae strains carrying no integrated plasmid (‘-‘), or with integrated plasmid pMH94 (vector) or pMM16 (with CarolAnn genes *43* and *44*), as indicated at the left; key plasmid features are indicated on the right. Liquid cultures of the plasmids were serially diluted 10-fold (right to left) and spotted onto solid media in the absence (‘Uninduced’) or presence (‘Induced’) of Atc inducer. (B) Growth inhibition by expression of Kita gp53 and Nymphadora gp53. Overnight cultures of G. terrae strains expressing CarolAnn genes *43* and *44* (plasmid pMM16) and either vector (pCCK38) or plasmids expressing Kita 53 (pMM63), Nymphadora *53* (pMM60), or the DEM 2C mutant of Nymphadora *53* (pM61) were subcultured into liquid medium at time zero and grown at 37°C. ATc inducer was added to each culture after 4 h (vertical arrow), and cell density (OD_600_) was measured every 2 h. (C) A model for CarolAnn *43*/*44*-mediated defense. CarolAnn gp43 is proposed to be membrane located but inactive as an ion channel until infection with phage Kita (or relatives). During early lytic growth of Kita, gp53 acts either directly or indirectly through CarolAnn gp44 to activate the gp43 ion channel, leading to loss of membrane potential and of intracellular ATP, interruption of macromolecular synthesis, and loss of cell viability.

Characterization of the defense escape mutants showed that CarolAnn genes *43* and *44* do not specifically inhibit an essential lytic function, and we therefore asked whether Kita *53* (and homologues) promotes cellular toxicity in the presence of CarolAnn *43*/*44*. Kita gene *53* was cloned into an inducible expression shuttle plasmid that replicates in G. terrae 3612. Because of ambiguity in the translation start site assignment, both a shorter version of the gene (translation start codon at coordinate 36992, encoding a 113-residue product) and a longer version (translation start codon at coordinate 36911, coding for a putative 140-aa protein) were cloned into vector pCCK38 to give plasmids pMM63 and pMM53, respectively. These extrachromosomally replicating plasmids are under Tet-ON control, with expression inducible with anhydrotetracycline (ATc). These were transformed into G. terrae 3612 strains carrying either integration-proficient plasmid vector pMH94 (43) or pMM16 expressing CarolAnn genes *43* and *44* (Fig. 4). Transformants were grown in liquid cultures, and serial dilutions were plated on solid media either with or without ATc inducer and incubated to test for viability following Kita *53* expression (Fig. 7A). Kita *53* expression from either plasmid caused no obvious growth defect under conditions of induction in the absence of CarolAnn *43*/*44* but showed reduced viability in the presence of CarolAnn *43*/*44*, with pMM63 having a somewhat stronger negative effect than pMM53. This suggests that the 113-aa product is active and that it is toxic to G. terrae when CarolAnn *43* and *44* are expressed, consistent with defense being mediated by cell death and the inability to complete all steps of viral production through the final lysis stage.

We similarly cloned and expressed Nymphadora *53* (Fig. 7A), as well as a single amino acid-substituted DEM mutant (Nymphadora_2C; Table 2). Only the longer version of the gene was cloned, and the results showed a strong negative growth impact under conditions of expression with CarolAnn *43*/*44* (Fig. 7A). Interestingly, the DEM mutant plasmid (pMM61) was not toxic, further supporting the idea that CarolAnn 43/44-dependent toxicity is associated with viral defense. We also determined the kinetics of growth inhibition in liquid cultures by inducing expression of Kita *53*, Nymphadora *53*, or the Nymphadora_2C DEM allele of *53* in mid-logarithmic growth (Fig. 7B). Induction of Kita *53* or Nymphadora *53* expression resulted in growth inhibition shortly after induction, suggesting that growth inhibition occurs in lytic growth within a time frame between early gene expression and completion of lytic growth (∼3 h).

To further analyze Kita gp53, we isolated a series of mutant derivatives of plasmid pMM53 from cells that overcame the toxicity and grew well in the presence of CarolAnn *43* and *44*. Eight independent mutants were isolated and the mutations identified (Fig. 6B). Each had a single amino acid substitution within the gene *53* open reading frame, two of which affected the same codon as the DEM mutations (Fig. 6A). One of these introduced a translational stop codon at position 80, presumably leading to loss of function (Fig. 6B). Taken together, these observations suggest that expression of Kita gp53 during infection of a CarolAnn lysogen contributes to loss of viability and abortive infection, strongly reducing lytic growth of Kita and related phages.

These observations are consistent with a model for CarolAnn 43/44 defense that is similar to lambda *rexAB* exclusion of T4*rII* (Fig. 7C). We propose that CarolAnn gp43 is membrane located and can act as an ion channel but only when activated by CarolAnn gp44. CarolAnn gp44 is in turn activated by infection with Kita or one of its close relatives, presumably through expression of Kita gp53 and a direct interaction with CarolAnn gp44 and, perhaps, gp43. Activation of the gp43 ion channel then leads to loss of membrane potential, reduction in intracellular ATP concentrations, interruption of macromolecular synthesis, and aborted infection. This growth inhibition is recapitulated by induction of Kita 53 expression in the presence of CarolAnn gp43 and gp44.

### Sbash genes *30* and *31* also defend against Kita and related *Gordonia* phages.

**FIG 8:**
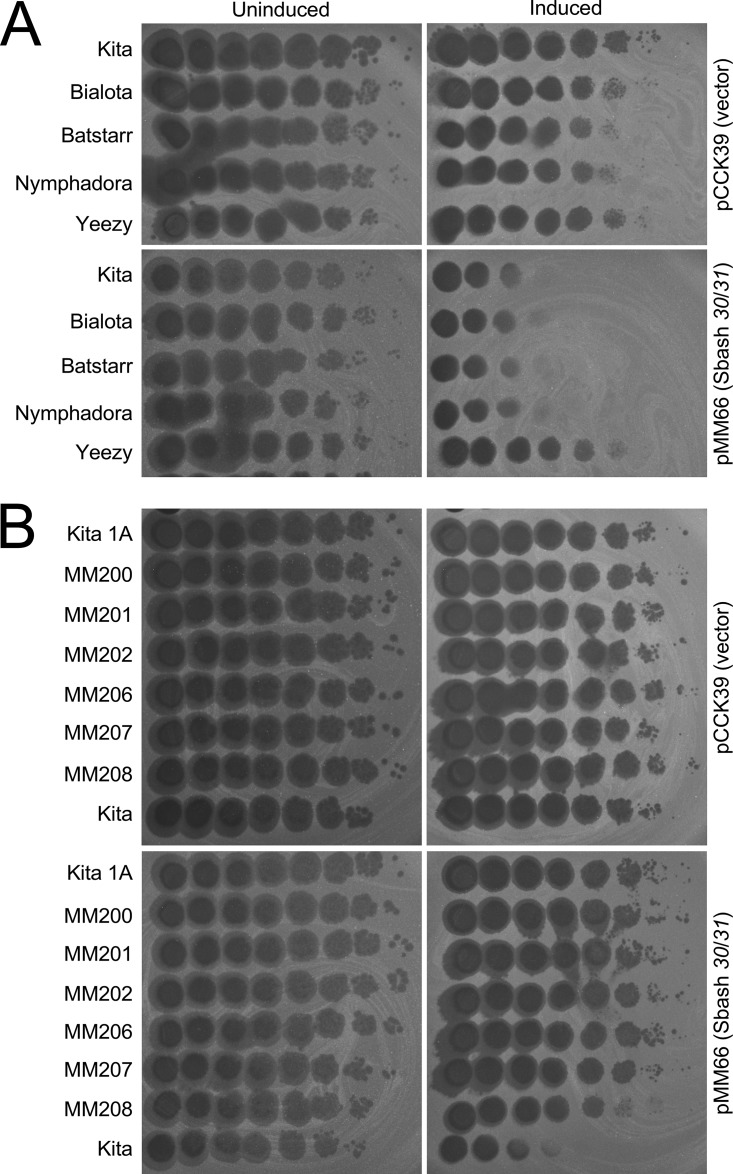
Sbash genes *30* and *31* confer defense against *Gordonia* phages. (A) *Gordonia* phages (as indicated on left) were serially diluted 10-fold and spotted onto lawns of *Gordonia* strains (as indicated on the right) carrying either plasmid vector pCCK39 or plasmid pMM66 with inducible Sbash genes *30* and *31*. Uninduced, solid media with no ATc inducer; Induced, solid media with ATc. Under conditions of induction, Sbash genes *30* and *31* confer defense patterns with respect to these phages that are similar to those seen with a strain expressing CarolAnn genes *43* and *44* (see Fig. 2). (B) Isolates of defense escape mutants against CarolAnn *43*/*44* defense also escape Sbash *30*/*31* defense. Ten-fold serial dilutions of seven DEMs (see Table 2) and wild-type Kita (bottom) were spotted on lawns of *Gordonia* strains as described above.

Mycobacteriophage Sbash carries genes *30* and *31*, which currently are the only other homologues of CarolAnn genes *44* and *43* in the Actinobacteriophage database (37). Sbash genes *30* and *31* defend specifically against mycobacteriophage Crossroads, which is unrelated to Kita and other *Gordonia* phages and does not have a homologue of Kita *53* (see accompanying manuscript [41]). Surprisingly, when Sbash genes *30* and *31* cloned downstream of an inducible promoter were expressed in G. terrae, they conferred defense against cluster CZ phages similarly to CarolAnn *43*/*44*, mirroring the strong defense against Kita, Bialota, Batstarr, and Nymphadora and the weaker defense against Yeezy (Fig. 2) (Fig. 8A). Furthermore, defense escape mutants isolates against CarolAnn 43/44 all also escaped Sbash 30/31 defense (Fig. 8B). In a reciprocal experiment, it was shown that pMM16 (expressing CarolAnn genes *43*/*44*), when introduced into Mycobacterium smegmatis, was capable of defending against mycobacteriophage Crossroads, and Crossroads DEMs isolates against Sbash 30/31 also escaped CarolAnn *43*/*44* (see accompanying paper [41]). The CarolAnn and Sbash systems thus operate interchangeably, even though the specific genes required to activate the defense are distinct in the native contexts.

## DISCUSSION

*Gordonia* temperate phages encode a variety of heterotypic viral defense systems with unpredictable patterns of defense. With the exception of phage UmaThurman, all of the cluster CV lysogens we tested conferred defense against infection by one or more heterotypic phages, and these may collectively have six or more distinct defense systems. This extends the repertoire of bacterial hosts that collude with their temperate phages to defend against viral attack beyond the *Pseudomonas* and *Mycobacterium* systems described previously (10, 11). However, the *Gordonia* and *Mycobacterium* systems share many common features. For example, the defense genes are located in the middle of the genomes, near the integration and immunity functions, and are expressed from the prophages in lysogeny. Characterization of these defense systems utilized the adaptation of mycobacterium-derived integration-proficient and extrachromosomal plasmid vectors for use in G. terrae, and these are likely to be generally useful for *Gordonia* genetics. Tet-ON inducible gene expression systems further add to the *Gordonia* genetic toolbox.

The CarolAnn system differs from the mycobacteriophage systems described previously in that the two required genes (*43*, *44*) are cotranscribed with the immunity repressor (*45*), an organization that is similar to that of the lambda *cI*-*rexA*-*rexB* system (Fig. 3B). We note that both phage Blueberry and phage Utz contain prophage-expressed genes (Fig. 3A), but these were not coexpressed with their respective repressor genes. It is also noteworthy that *Mycobacterium* phage Sbash genes *30* and *31* are homologues of CarolAnn *44* and *43* and that these are also not coexpressed with the Sbash repressor (gene *43*). There is thus considerable variation in how the defense genes are organized and expressed in the prophage genomes.

The CarolAnn defense system is heterotypic and specifically targets phages carrying homologues of Kita *53* in cluster CZ and in subcluster CY1; phages such as Howe lacking a Kita *53* homologue are not targeted for defense (Table 1). The role of Kita *53* in phage growth is unclear, although it is expressed in early lytic growth and does not appear to be required for phage propagation. There are few bioinformatic clues to its function, and it is therefore unclear why Kita and the related phages would carry a gene that places it at a disadvantage with respect to being targeted by the CarolAnn defense. A plausible explanation is that it plays a role similar to that described for gp52 encoded by mycobacteriophage Fruitloop, which excludes superinfection of other phages during Fruitloop lytic growth but is not required for lytic growth *per se* (44). In this scenario, carrying a gene such as *53* represents a double-edged sword for Kita, in that it may fend off competing phages in one scenario, while rendering it subject to inhibition by others.

A simple mechanistic explanation for CarolAnn *43*/*44* defense is that Kita gp53 activates a growth defect that results in loss of viability. Although a similar phenomenon has been described for toxin-antitoxin-mediated abortive infection (9), the CarolAnn system is not evidently a TA system, in that expression of CarolAnn gp43 or gp44 alone is not toxic (Fig. 4). However, expression of Kita gp53 is clearly toxic in the presence of CarolAnn gp43 and gp44, and the finding that defense escape mutant derivatives of gp53 lose this toxicity supports the interpretation that cytotoxicity is mechanistically related to viral defense. Expression of CarolAnn genes *43* and *44* does not alter the transcriptomic patterns in Kita infection, and it is plausible that phage development is mostly unaffected. Because of the similarities of CarolAnn *43*/*44* to lambda *rexAB*, an attractive explanation is that Kita gp53 and CarolAnn gp44 activate membrane protein CarolAnn gp43 with respect to its ability to depolarize the cellular membrane potential, reduce intracellular ATP concentration, and interrupt macromolecular synthesis.

The only sequenced actinobacteriophage with homologues of CarolAnn gp43 and gp44 is mycobacteriophage Sbash; CarolAnn gp43 and gp44 and Sbash gp31 and gp30 share 42% and 50% amino acid identity, respectively. Curiously, Sbash *30*/*31* defends against phage Crossroads attack, and escape mutants map in both Crossroads *132* and Crossroads *141*, which are unrelated to Kita *53* and are absent from any of the *Gordonia* phages. Remarkably, when Sbash genes *30* and *31* are expressed in *Gordonia*, they defend against Kita and other CV phages (Fig. 8), and when CarolAnn genes *43* and *44* are expressed in M. smegmatis, they defend against Crossroads (see accompanying paper [41]). In both instances, DEMs isolated in the cognate context also escape the hybrid context. These observations illustrate the complexities of the defense systems and the specificities with which they target attacking phages. We envisage that these complexities result from powerful red queen dynamic interactions between phages and their hosts, strong host selection for protection against viral attack, and the success of viral derivatives that escape the defense system but likely do so at the expense of other functionalities (such as exclusion) that confer selective advantages under conditions of attacks by different sets of phages.

## MATERIALS AND METHODS

### Phage growth and analysis.

High-titer phage lysate was generated by serially diluting phage samples into phage buffer with 2 mM calcium chloride by 10-fold increments. Dilutions were plated using 10 µl of phage dilution, 3.5 ml PYCa top agar, and 250 µl G. terrae ATCC 3612 on PYCa solid agar plates supplemented with calcium chloride (33). Petri plates were incubated for 48 to 72 h at 30°C until confluent infection was observed. Plates were flooded with 5 ml of phage buffer (2 mM CaCl_2_) and were incubated for 4 h at room temperature or for 12 to 24 h at 4°C. Floods were harvested by the use of a syringe and a 0.22-µm-pore-size filter and were stored at 4°C for use. Genomic analyses were performed using the Phamerator database (Actinobacteriophage_Draft; August 2018) (45). Phage genomic DNA was isolated using phenol-chloroform/isoamyl alcohol extraction followed by isopropanol/sodium acetate precipitation (46) and was sequenced using Illumina MiSeq 150 base single-end runs and assembled as described previously (47).

### Strains and plasmids.

Gordonia terrae 3612 was used to isolate and characterize phages (40), and phage lysates typically had titers of >10^9^ PFU ml^−1^. Lysogens (serially diluted 10-fold) were isolated on spot titer plates, and purification of bacterial survivors on PYCa solid agar was performed; isolated colonies were patched on G. terrae 3612 lawns to confirm phage release, and lysogens were confirmed to be immune to superinfection.

Oligonucleotide primers and plasmids are described in Tables S1 and S2 in the supplemental material. Recombinant plasmids pMM15, pMM16, pMM59, and pMM18 were constructed using a phage L5 integration cassette containing pMH94 with a kanamycin resistance marker (43). Gibson assembly was used to ligate PCR-amplified insertions into the EcoRI linearized pMH94 backbone. Vector pCCK38 (44) is an extrachromosomal plasmid with a hygromycin resistance marker and *oriE* and *oriM* replication origins for propagation in Escherichia coli and mycobacteria (and G. terrae), respectively. The pCCK38 vector was used to generate pMM53, pMM63, pMM60, and pMM61. Plasmids pMM55, pMM56, and pMM57 are derivatives of pCCK39 (44), an integrative version of pCCK38, containing *oriE* for propagation in E. coli, and the phage L5 integration cassette (43). Plasmid pMM37 is an extrachromosomal plasmid derivative of pCCK38 containing a kanamycin resistance marker. Plasmids pCCK38, pCCK39, and pMM37 contain the Tet-ON system for inducible expression of genes by addition of anhydrotetracycline (Atc) (48) and were linearized by PmlI restriction digestion for ligation of PCR-amplified insertions. Inserts for all plasmids were PCR amplified using Q5 high-fidelity DNA polymerase (New England BioLabs, Inc.), appropriate primers (see Table S1), and high-titer phage lysate as the template. All plasmids were confirmed by Sanger sequencing (Genewiz). Mutant derivatives of pMM16, pMM59, pMM18, and pMM63 were made using a Q5 site-directed mutagenesis kit (New England BioLabs, Inc.).

10.1128/mBio.02417-18.4 TABLE S1 Oligonucleotide primers used in this study. Download Table S1, DOCX file, 0.01 MB. Copyright © 2019 Montgomery et al. 2019 Montgomery et al. This content is distributed under the terms of the Creative Commons Attribution 4.0 International license.

10.1128/mBio.02417-18.5 TABLE S2 Plasmids constructed in this study. Download Table S2, DOCX file, 0.01 MB. Copyright © 2019 Montgomery et al. 2019 Montgomery et al. This content is distributed under the terms of the Creative Commons Attribution 4.0 International license.

### Electroporation of Gordonia terrae.

Plasmids were introduced into G. terrae by electroporation using electrocompetent G. terrae cells, prepared by growth of 50-ml cultures in Middlebrook 7H9 media supplemented with ADC for 48 to 72 h to an optical density at 600 nm (OD_600_) between 0.7 and 1.0, and centrifuged at 6,000 relative centrifugal force (rcf) for 10 min. Gordonia terrae has a density of ∼1.0 × 10^8^ CFU ml^−1^ at an OD_600_ of 0.5. Cell pellets were washed three times with ice-cold buffer (10% glycerol, 250 mM sucrose, 0.5 mM potassium acetate) and resuspended in 1 ml of buffer; 100-µl aliquots were stored at −80°C. Electroporations were performed using 100 ng DNA and 100 μl cells in 0.2-cm-path-length electrode gap cuvettes (Bio-Rad) at settings of 2,500 V, 1,000 Ω, and 25 µF. Cells were recovered in 1 ml of 7H9 Middlebrook medium with ADC for 2 h shaking at 250 rpm. Transformants were recovered on LB agar (Difco Laboratories) plates supplemented with appropriate antibiotics.

### Immunity assays.

Lysogenic cultures were inoculated from single colonies into PYCa broth (33) that included 5 to 10 sterile glass beads and were grown for 48 to 72 h at 30°C. If necessary for cell dispersal, cultures were mildly sonicated (intervals of 15 s on and 10 s off, 10 min total, 30% amplitude), and 0.25 ml of culture was plated with 3 ml of PYCa top agar (3.25% agar) (33). Phage lysates were serially diluted 10-fold, and a 3-µl volume was spotted onto bacterial lawns. Plates were incubated for ∼72 h at 30°C.

### Isolation and characterization of defense escape mutants (DEMs).

Escape mutant plaques were picked from bacterial lawns, purified, and amplified on G. terrae 3612. Mutant phenotypes were confirmed by plating dilutions of putative mutants on G. terrae 3612 and the CarolAnn lysogen or pMM16. Genomic DNA was extracted and sequenced as described previously (47).

### RNAseq analysis.

RNA was isolated from phage-infected cultures at an OD_600_ of ∼0.8 (multiplicity of infection [MOI] = 3.0) at 30 min and 120 min postinfection. Cultures were lysed with TRIzol reagent (Gibco/BRL) (10-min incubation) followed by four rounds of bead beating. RNA samples were DNase treated with Turbo DNA-free (Ambion), and rRNA was depleted using Ribo-Zero kits (Illumina); cDNA library preparation was performed using a TruSeq stranded RNAseq kit (Illumina). The fastq reads were analyzed as described previously (10), and was coverage viewed and results presented with the Integrative Genomics Viewer (49). RNAseq data sets, with additional method details, have deposited in the Gene Expression Omnibus (GEO) with accession number GSE121959.

### Toxicity assays.

Gordonia terrae 3612 strains were grown to mid-log phase in liquid (5 ml) with appropriate selective antibiotics, where appropriate, and adjusted to an OD_600_ of 0.5. Cultures were serially diluted 10-fold, and a 2.5-µl volume of the dilutions was plated on solid medium with or without inducer (Atc, 100 ng/ml); antibiotics were included where appropriate. Plates were incubated at 30°C in the absence of light for 3 days.

### Recombinant plasmid mutagenesis.

Plasmids were transformed into competent E. coli XL1-red cells (Agilent Technologies), plated, and incubated overnight at 37°C. Plates were flooded with 5 ml of LB broth (Difco Laboratories) and incubated for 10 min at room temperature. The culture was recovered and grown overnight at 37°C (250 rpm). DNA was extracted and transformed into a CarolAnn lysogen cells by electroporation. Isolated colonies were picked and electroinduced in electrocompetent E. coli XL1-Blue cells (Agilent Technologies) (2,500 V, 100 Ω, 25 µF) (50). Isolated colonies were picked and grown in selective LB broth (Difco Laboratories), and plasmid DNA was extracted and sequenced.

### Data availability.

All phage genome sequences are available at https://phagesdb.org. The GenBank accession numbers are listed in Table 1. RNAseq data sets, with additional method details, have deposited in the Gene Expression Omnibus (GEO) with accession number GSE121959.
