# Supplementary figures and images for: Yet More Evidence of Collusion: a New Viral Defense System Encoded by Gordonia Phage CarolAnn
Source: mBio. 2019 Mar 19;10(2):e02417-18. doi: 10.1128/mBio.02417-18 (PMC6426606; doi:10.1128/mBio.02417-18)

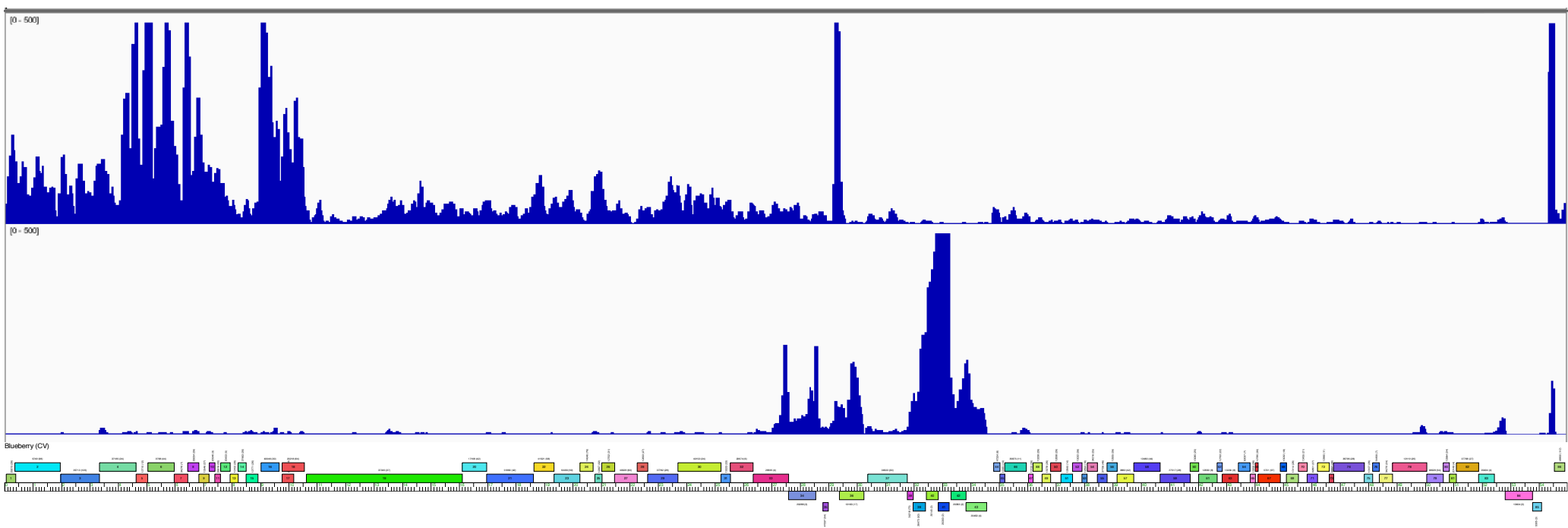

Figure S2

Supplement: FIG S2 [file mBio.02417-18-sf002.pdf]

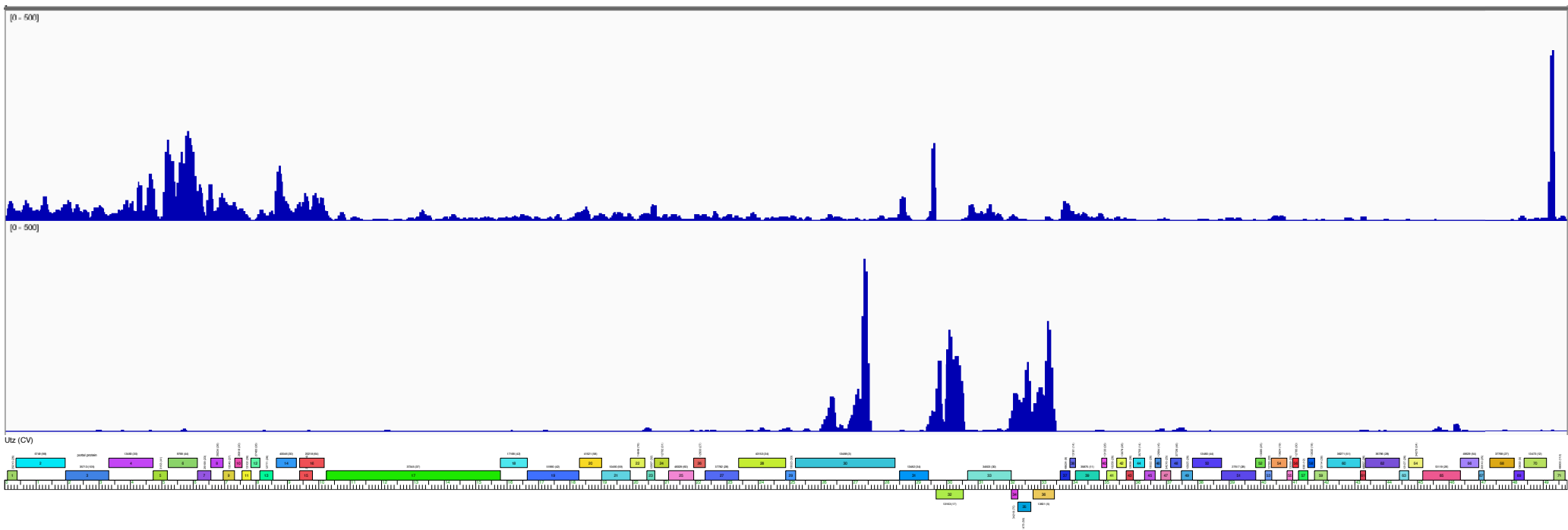

Supplement: FIG S3 [file mBio.02417-18-sf003.pdf]
