# Supplementary material for: Yet More Evidence of Collusion: a New Viral Defense System Encoded by Gordonia Phage CarolAnn
Source: mBio. 2019 Mar 19;10(2):e02417-18. doi: 10.1128/mBio.02417-18 (PMC6426606; doi:10.1128/mBio.02417-18)
Supplement: TABLE S1 [file mBio.02417-18-st001.docx]

Table S1. Oligonucleotide primers used in this study

| **Primer** | **Sequence** |
| --- | --- |
| pMH94 flanking FWD | ACTATGGTTGCTTTGACGTGCGGTGTGAAA |
| pMH94 flanking REV | GGAGCTGGTGCAGTGAAGAGAATAGACCG |
| CA43_44_45 FWD | AAAACGACGGCCAGTGAATTCTCCAACTTCCCTTCGGCC |
| CA43_44_45 REV | CGGGTACCGAGCTCGAATTCTCAACTGACCTGTCTGAGC |
| SDM pMM16 FWD  Mm1 | GAAGAATCGCAAGAGCAAG |
| SDM pMM16 REV | ACTCATGGATTGAGGTTC |
| SDM pMM17 FWD | GAATTCGAGCTCGGTACC |
| SDM pMM17 REV | TCAATTCGCTTCCGAGCC |
| SDM pMM18 FWD | GTGATCAGGGCTCGGAAG |
| SDM pMM18 REV | CAGTCGTTCTCCTCTTGC |
| Kita_gp53_RBS FWD | ATCACAGCTGCAGAATTCGAAGCTTGCGTCGAACACGGCCAGA |
| Kita_gp53_RBS FWD | TAACTACGTCGACATCGATAAGCTTTCAGTTGCCTTCCGGGGC |
| pMM53 FWD | GATTCGCCGCCCGAAATCACGTGGCGTCGAACACGGCCAGA |
| pMM53 REV | GCGTTTAAACCTGCAGGCACGTGTCAGTTGCCTTCCGGGGC |
| pMM55 FWD | GATTCGCCGCCCGAAATCACGAATCGCAAGAGCAAGAG |
| pMM55 REV | GCGTTTAAACCTGCAGGCACTCAACTGACCTGTCTGAG |
| pMM56 FWD | GATTCGCCGCCCGAAATCACGAATCGCAAGAGCAAGAG |
| pMM56 REV | GCGTTTAAACCTGCAGGCACTCAATTCGCTTCCGAGCC |
| pMM57 FWD | GATTCGCCGCCCGAAATCACAGGTCAAGTCCGATGCCC |
| pMM57 REV | GCGTTTAAACCTGCAGGCACTCAACTGACCTGTCTGAGC |
| pMM60-61 FWD | GATTCGCCGCCCGAAATCACGGTGAACCAGTGCCGCTG |
| pMM60-61 REV | GCGTTTAAACCTGCAGGCACTCAGTTGCCTTCCGGGGC |
| pMM63 FWD | GATTCGCCGCCCGAAATCACGTGGAAGGGAAAGTCAGTGACCGAC |
| pMM63 REV | GCGTTTAAACCTGCAGGCACGTGTCAGTTGCCTTCCGGGGC |
