# Supplementary material for: Yet More Evidence of Collusion: a New Viral Defense System Encoded by Gordonia Phage CarolAnn
Source: mBio. 2019 Mar 19;10(2):e02417-18. doi: 10.1128/mBio.02417-18 (PMC6426606; doi:10.1128/mBio.02417-18)
Supplement: TABLE S2 [file mBio.02417-18-st002.docx]

Table S2. Plasmids used in this study

| **Plasmid** | **Vector** | **Phage Gene(s)** | **Phage Coordinates** |
| --- | --- | --- | --- |
| pMM15 | pMH94 | CarolAnn *43-45* | 35,464 – 38,066 |
| pMM16 | pMH94 | CarolAnn *43-44* | Derivative of pMM15 (Δ37,119 – 37,791) |
| pMM59 | pMH94 | CarolAnn *44* | Derivative pMM16 (Δ35,464 – 35,974) |
| pMM18 | pMH94 | CarolAnn *43* | Derivative pMM16 (Δ36,001 – 37,087) |
| pMM38 | pMM37 | Kita *53* | 36,881 – 37,333 |
| pMM53 | pCCK38 | Kita *53* | 36,881 – 37,333 |
| pMM55 | pCCK39 | CarolAnn *43-44* | 35,464 – 37,116 |
| pMM56 | pCCK39 | CarolAnn *44* | 35,975 – 37,116 |
| pMM57 | pCCK39 | CarolAnn *43* | 35,464 – 36,030 |
| pMM60 | pCCK38 | Nymphadora *53* | 38,865 – 39,296 |
| pMM61 | pCCK38 | Nymphadora DEM 2C *53* | 37,779 – 38,210 |
| pMM62 | pCCK39 | Kita *53* | 36,881 – 37,333 |
| pMM63 | pCCK38 | Kita *53* | 36,979 – 37,333 |
