# Supplementary material for: Yet More Evidence of Collusion: a New Viral Defense System Encoded by Gordonia Phage CarolAnn
Source: mBio. 2019 Mar 19;10(2):e02417-18. doi: 10.1128/mBio.02417-18 (PMC6426606; doi:10.1128/mBio.02417-18)
Supplement: FIG S1 [file mBio.02417-18-sf001.pdf]

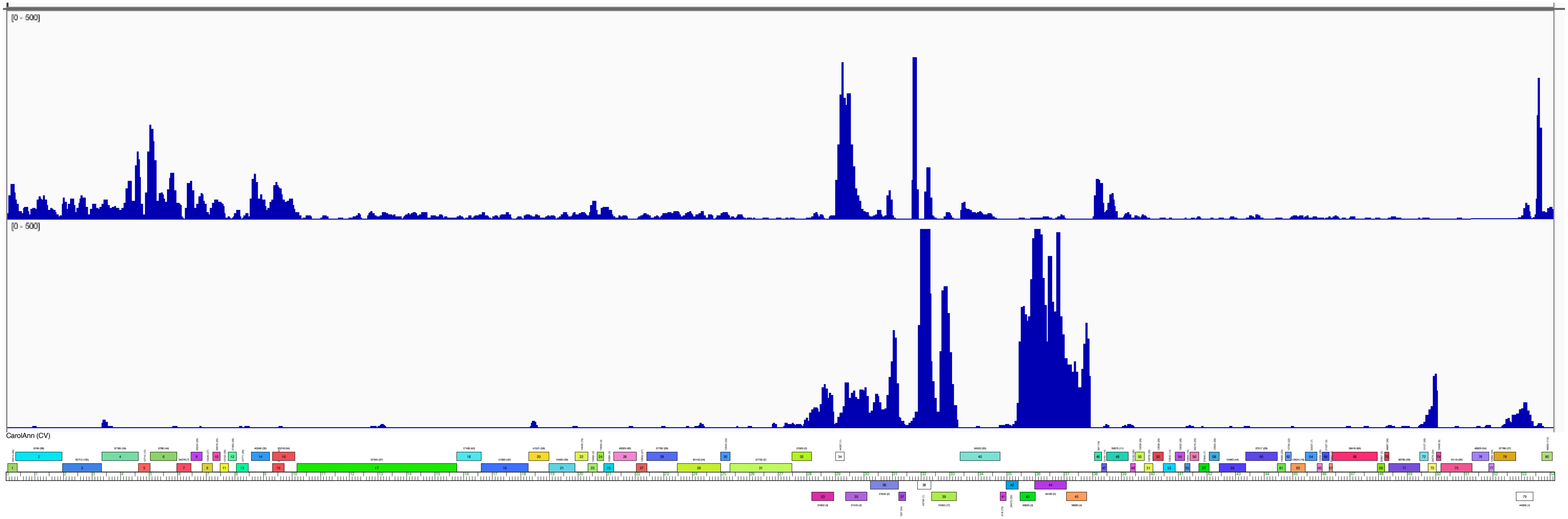

Figure S1
